# Novel ten second antimicrobial coating for endotracheal tubes to prevent ventilator associated pneumonia

**DOI:** 10.1038/s41598-025-31706-7

**Published:** 2026-01-12

**Authors:** Manar Fathy Al-Sayed, Mohamed Tarek El-Wakad, Mohammed A. Hassan, Ahmed M. Soliman, Amal S. Eldesoky

**Affiliations:** 1 Biomedical Engineering Department Faculty of Engineering , Helwan University , Cairo, Egypt; 2 Biomedical Engineering Department , Future University in Egypt , Cairo, Egypt; 3 Biomedical Engineering Department , Higher Technological Institute 10th of Ramadan , Cairo, Egypt

**Keywords:** Endotracheal tube, Ventilator-associated pneumonia, Antimicrobial coating, Dimethylformamide, Tetrahydrofuran, Polyvinyl chloride, Biocompatibility, Bacterial colonization, Escherichia coli (E. coli), Critical care, Insta-coating, Biomedical engineering, Biomaterials

## Abstract

This novel approach aims to mitigate ventilator-associated pneumonia (VAP) in critically ill patients by improving the coating of endotracheal tubes (ETT). The research focuses on creating durable, antimicrobial coatings to prevent bacterial colonization on endotracheal tube surfaces. Using a 10-second exposure to solvents such as dimethylformamide (DMF) and tetrahydrofuran (THF), the study successfully demonstrated a robust coating on polyvinyl chloride (PVC) tubing. This coating inhibits the growth of Escherichia coli while maintaining biocompatibility and avoiding the toxic effects of traditional silver nitrate coatings. Quantitatively, the coatings achieved pH values of 5–5.5, viscosities of 0.6-1 mPa·s, and reduced microbial counts to 4 CFU/µL, compared to 5 CFU/µL in the controls. Biocompatibility remained high, with cell viability exceeding 98% for DMF and THF coatings. The research highlights a significant advance in combating ventilator-associated pneumonia, although clinical trials are needed to validate these promising results for clinical applications in intensive care settings.

## Introduction

Ventilator-associated pneumonia (VAP) represents a formidable challenge in critical care, posing a considerable threat to the already vulnerable population of critically ill patients undergoing mechanical ventilation. Tracheal intubation, a fundamental procedure for ensuring unimpeded airflow in these patients, paradoxically amplifies the risk of VAP due to the colonization of bacterial pathogens along the surfaces of endotracheal tubes. This complication not only prolongs hospital stays but also markedly compromises patient outcomes, thereby necessitating urgent strategies to mitigate its impact^1–4^.

The quest to prevent VAP has spurred innovative research endeavors aimed at refining endotracheal tube coatings. These coatings serve as a potential frontline defense against microbes; however, current antimicrobial coatings for ETTs face limitations, including cytotoxicity of silver nitrate, rapid degradation of polymeric layers, and insufficient resistance to biofilm formation. These drawbacks highlight the urgent need for safer, more durable alternatives to colonization and subsequent respiratory infections. This study stands at the forefront of these efforts, dedicated to optimizing the in vitro coating process for endotracheal tubes. By seeking novel techniques to enhance the durability and efficacy of these coatings, the overarching objective is to curtail the incidence of VAP among ventilated patients, thereby revolutionizing medical interventions and patient care within intensive care units (ICUs)^5–7^.

In a comprehensive exploration encompassing a spectrum of substances, this research meticulously examines the interaction and efficacy of various materials. Assessments range from meticulous examinations of pH, viscosity, and antibacterial properties to intricate evaluations of effects on cell lines and biocompatibility. Leveraging innovative microscopic analyses such as scanning electron microscopy (SEM) and transmission electron microscopy (TEM), the study evaluates the efficacy and safety of the newly formulated coatings^8–11^.

The pivotal findings of this research showcase that a brief exposure of only 10 s to specific solvents, notably dimethylformamide (DMF) and tetrahydrofuran (THF), yields a stable and robust coating on polyvinyl chloride (PVC) tubes. This coating demonstrates potent antimicrobial properties, effectively restraining the proliferation of Escherichia coli (a Gram-negative bacterium), while avoiding the toxic repercussions associated with silver nitrate. Crucially, the coating exhibits exceptional biocompatibility and portrays promising results under SEM and TEM analyses^8,11–15^.

This study builds upon prior efforts to mitigate ventilator-associated pneumonia (VAP) through innovative endotracheal tube (ETT) coatings. Previous approaches have included noble metal coatings, such as silver or gold, which demonstrated delayed VAP onset but raised concerns about cytotoxicity and inconsistent clinical benefit^16–18^. Hydrogel-based polymeric coatings have also been explored, offering controlled drug release but requiring longer preparation times and showing limited durability under mechanical stress^19^. More recently, nanocomposite coatings have been proposed to enhance antifouling and biofilm resistance, yet these methods often involve complex fabrication steps and extended exposure periods^20^. Meta-analyses of metal-coated ETTs confirm modest reductions in VAP incidence but highlight variability in outcomes and safety concerns^21^. In contrast, our work introduces a rapid 10-second solvent-based coating using DMF and THF, which achieves comparable antibacterial efficacy against E. coli while maintaining superior biocompatibility (> 98% cell viability). This incremental advance lies in the **combination of speed**,** simplicity**,** and safety**, offering a practical alternative to existing strategies and addressing limitations of cytotoxicity, durability, and clinical feasibility.

This study heralds a groundbreaking advancement in endotracheal tube coating technology, offering a promising strategy to curtail the incidence of VAP among ventilated patients in critical care settings. The imminent translation of these in vitro findings into real-world applications holds the potential to significantly transform patient care, fortify medical interventions, and substantively mitigate the burden of VAP in intensive care scenarios. However, further research through clinical trials is still needed to ensure that these promising laboratory results serve as the basis for effective medical interventions^8,12–15^.

## Materials and methods

### Material used

Silver nitrate sol-gel (AgNO_3_), sodium hydroxide (NaOH), distilled water (H_2_O), Acetic acid (CH₃COOH), Toluene (TOL), Tetrahydrofuran (THF), Dimethylformamide (DMF), and Polyvinyl chloride (PVC) with Molecular Weights 169.873, 40.01, 188.015, 60.052, 92.14, 72.11, 73.09, and 233,000 g/mol respectively were supplied by Egyptian chemical manufacturers. Endotracheal Tube (ETT) Cuffed/Size 7.5 mm-L 28 cm/Dimensions 18 × 3 × 0.25 in were supplied by ULTRA MED for the medical products company, in Egypt. Concentration and stirring Time are shown in Table (1).

**Table 1:** Amount of all materials.

Experiments	AgNO_3_ Concentrations	NaOH Concentrations	Stirring Time (Min)	TOL (mL)	THF (mL)	DMF (mL)	PVC (gm)
1 st Experiment	0.1852%	4%	30	50	-	-	1
2nd Experiment				-	25	-	
3rd Experiment				-	-	25	

### Mixture preparation

Silver nitrate Sol-Gel was prepared by dissolving silver nitrate and sodium hydroxide in distilled water, and stirring for sixty minutes at 45 °C. Then, acetic acid was added drop by drop and stirred again^22^.

Then, in sample (1), we add a mixture of 1gm of PVC dissolved in Toluene. In sample (2), we added a mixture of 1gm of PVC dissolved in THF. In sample (3), we add a mixture of 1gm of PVC dissolved in DMF. All experiments are stirred for thirty minutes at 50 °C.

The solvents were selected based on their differential solubility of PVC and their influence on coating morphology. THF and DMF are strong PVC solvents, enabling uniform film formation, while toluene provides a weaker solubility baseline for comparison.

**Fig. 1:**
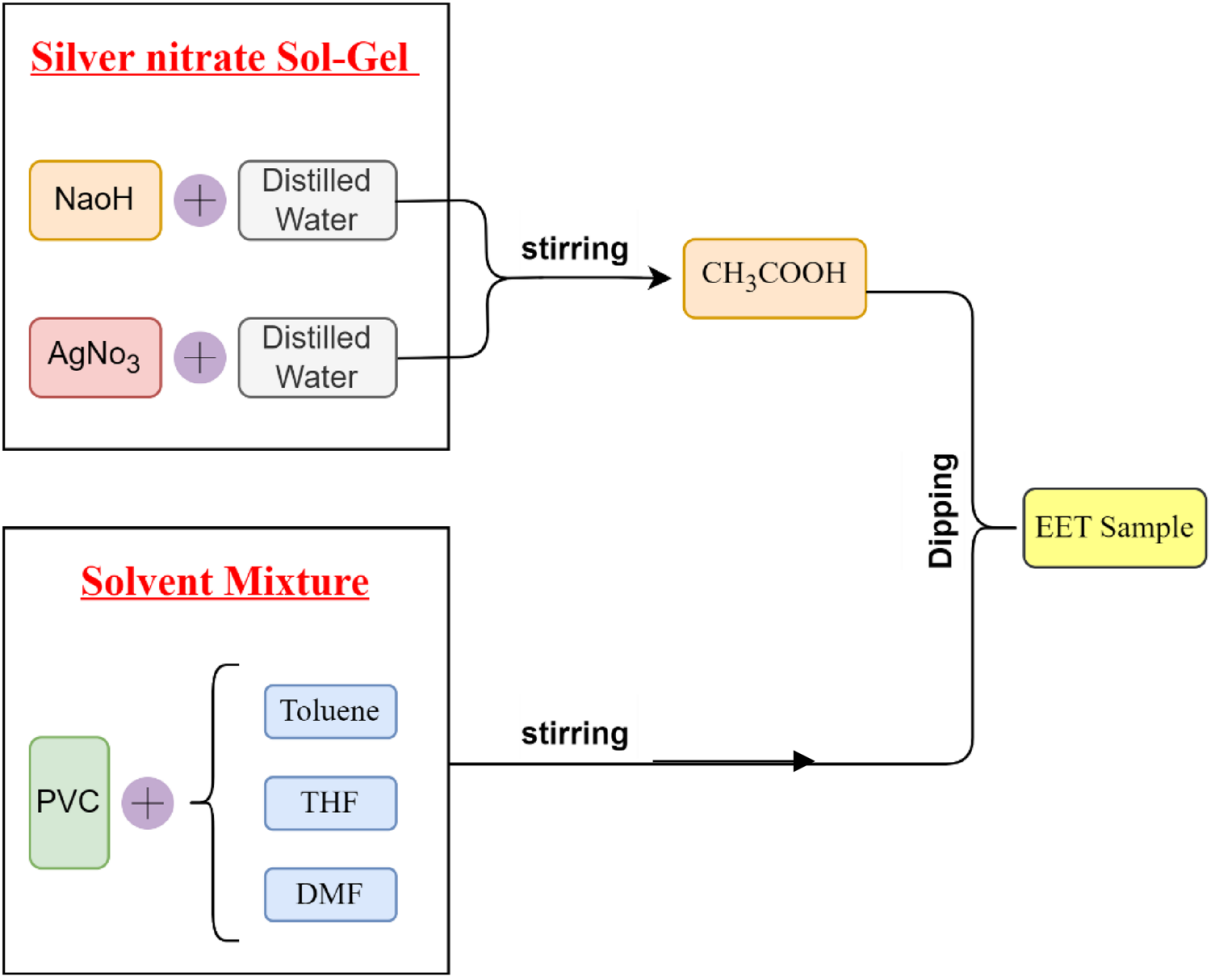
Prepared sequence.

Finally, we add the silver nitrate Sol-Gel mixture to each sample and stirred for 60 min, then the ETT samples were dipped in the solution according to the sequence shown in Figure (1) and coated for 10 s.

After dipping, samples were gently rinsed with distilled water to remove unbound residues and air-dried at room temperature for 30 min. This step ensured stabilization of the antimicrobial layer before testing.

Uncoated ETTs were included as negative controls in all experiments. Each test condition was performed in triplicate (*n* = 3), and results are reported as mean ± standard deviation.

**Fig. 2:**
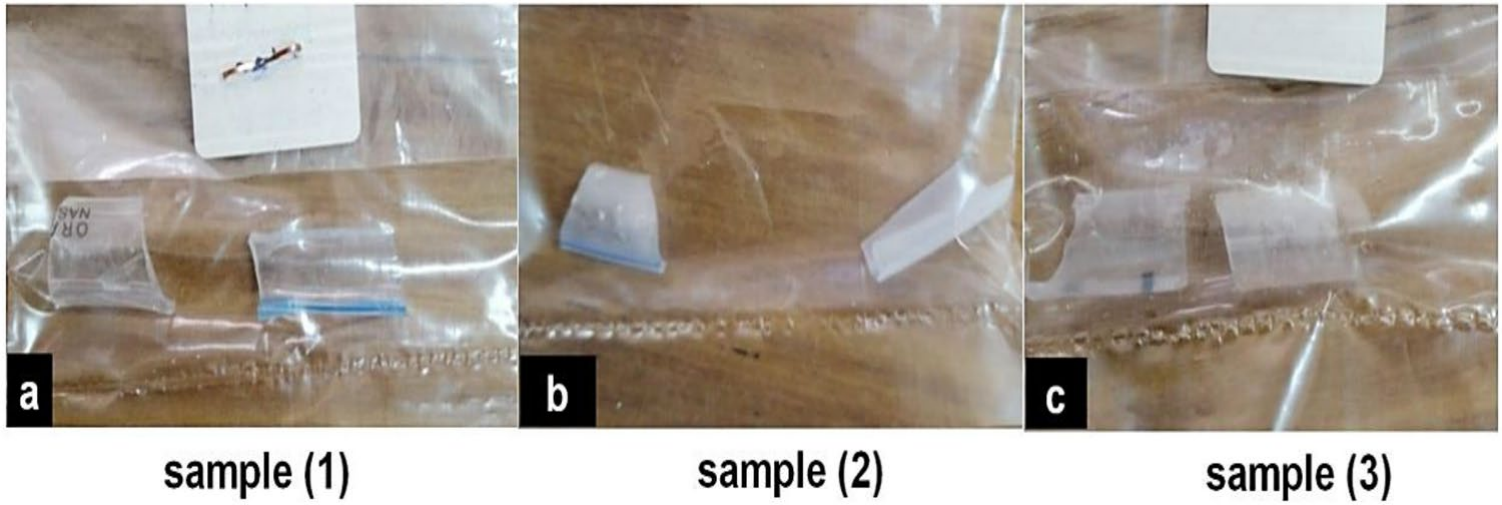
The dipping samples: (**a**) Toluene, (**b**) THF, (**c**) DMF.

Samples 1, 2, and 3, as shown in Fig. 2, provide a comprehensive visual representation of the observed differences, highlighting the distinctive characteristics of each. The coating thickness is shown to be medium for toluene, greatest for DMF, and maximum for THF.

**Testing Phase**:

During this phase, a thorough and detailed assessment was conducted to examine the attributes and potential of the developed coating. This evaluation encompassed a multifaceted approach, involving critical tests: pH analysis, viscosity measurement, examination of antibacterial effects, and assessment of biocompatibility.

**pH Analysis**:

A digital pH meter (PH-200), with a measurement range of 2–10 pH units and an accuracy of ± 0.01 pH units, was used to measure the pH of each solution during the experiments. The temperature was consistently maintained at 50^°^C, and the meter was calibrated using standard buffer solutions before each measurement. Regular pH measurements were recorded to monitor any changes over time. These measurements were crucial for evaluating the impact of different experimental conditions on the solutions’ pH and their effectiveness in preventing bacterial colonization and VAP in ETT samples.

**Viscosity Measurement**:

A digital rotational viscometer (DRV), specifically the VSC-E1 Series analyzer, was employed to analyze the viscosity of the AgNO_3_ Sol-Gel experiment. This DRV analyzer could measure viscosity at shear rates below 10s^⁻¹^, making it ideal for evaluating the solution’s performance in dipping and coating applications. It required a small sample size of 0.5 mL and operated within a temperature range of 50^°^C. The analyzer boasted an accuracy of at least ± 1% and a repeatability precision of at least ± 0.5%, ensuring precise and reliable measurements. Viscosity measurements were taken at various time intervals during the experiment to determine the optimal conditions for preventing bacterial colonization and VAP in the endotracheal tube (ETT) samples.

**Fig. 3:**
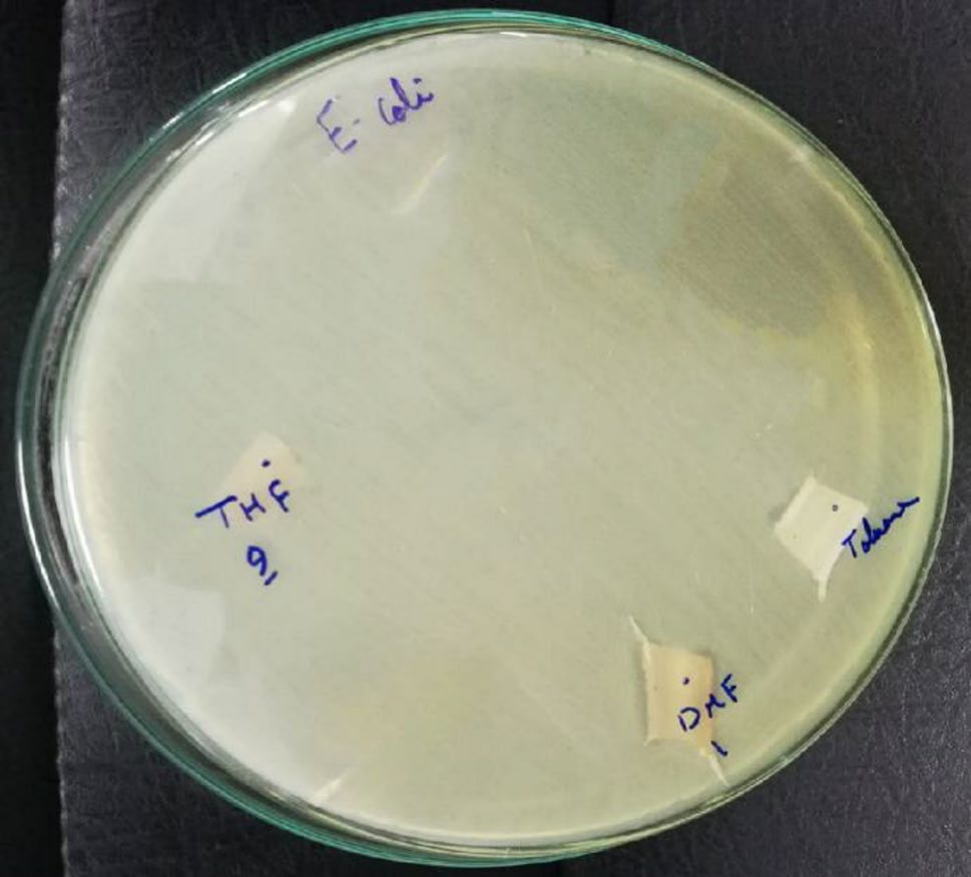
Gram-Negative Bacteria: Escherichia coli ATCC 25,922 for all samples.

**Assessment of Antibacterial Effects**:

Extensive tests were performed to assess the coating’s effectiveness in inhibiting bacterial growth. In this study, Gram-negative E. coli ATCC 25,922 was used in each sample to evaluate the solutions’ ability to prevent bacterial colonization in ETT samples, as illustrated in Figure (3). This strain was chosen due to its well-characterized genome sequence, non-pathogenic nature, extensive research in disease physiology, and its utility as a model for studying specific antibiotics and systems in Gram-negative bacteria. CFU quantification was performed by serial dilution and plating on nutrient agar, followed by incubation at 37 °C for 24 h. Colonies were counted manually and expressed as CFU/µL. Its high manufacturing quality and suitability as a reference or control strain make it an ideal choice for this study. Statistical analysis was performed using one-way ANOVA with Tukey’s post-hoc test. A p-value.

### Examination of biocompatibility

The study meticulously examined the coating’s biocompatibility, which is essential for its safe interaction with living tissues or cells. This involved evaluating the coating’s effects on normal cell lines to ensure it did not negatively impact cellular viability or function, thereby confirming its safety for potential medical applications.

Each of these tests was crucial in thoroughly evaluating the coating’s properties, aiming to develop a formulation that not only effectively prevents bacterial colonization but also ensures compatibility and safety for use in critical care settings. This comprehensive evaluation highlighted the study’s commitment to creating a protective coating with optimal functionality and biocompatibility, potentially marking a significant advancement in preventing ventilator-associated pneumonia^23–26^.

Biocompatibility was assessed using normal human fibroblast (NHF) cells cultured in DMEM supplemented with 10% FBS and 1% penicillin‑streptomycin. Cell viability was quantified using the MTT assay, with absorbance measured at 570 nm. Representative images of cell morphology were captured using an inverted microscope.

Imaging Techniques:

In the methodology phase, advanced imaging techniques, specifically Scanning Electron Microscopy (SEM) and Transmission Electron Microscopy (TEM), were employed to explore and analyze the structural and morphological attributes of the developed coatings.

### Scanning electron microscopy (SEM)

Scanning Electron Microscopy (SEM) is a powerful technique for obtaining high-resolution images of a sample’s surface. The process involves several steps: preparing the sample, directing an electron beam onto it, detecting the resulting signal, and processing the image. In this study, the SEM (JSM-IT200) settings were optimized to ensure high-quality images with sufficient resolution. SEM analysis enabled the visualization of the surface morphology of the ETT samples after treatment with various solutions, providing insights into the coating’s effectiveness in preventing bacterial colonization and ventilator-associated pneumonia (VAP). These images were essential in evaluating the coating’s potential as a preventative measure against VAP in critically ill patients.

### Transmission electron microscopy (TEM)

Transmission Electron Microscopy (TEM) is a powerful technique for obtaining high-resolution images of internal structures at the nanoscale. The TEM analysis process involves passing an electron beam through a thin sample, detecting the resulting signal, and creating an image of the structure. The TEM settings used included an accelerating voltage of 120 kV, a beam current of 200 nA, and a detector for bright-field or dark-field images. TEM analysis enabled the visualization of the internal structures of the ETT samples after treatment with various solutions, providing insights into the coating’s effectiveness in preventing bacterial colonization and ventilator-associated pneumonia (VAP). The high-resolution images obtained through TEM offered valuable information about structural changes in the ETT samples post-treatment^27–30^.

## Results and discussion

**pH analysis result:**

**Fig. 4:**
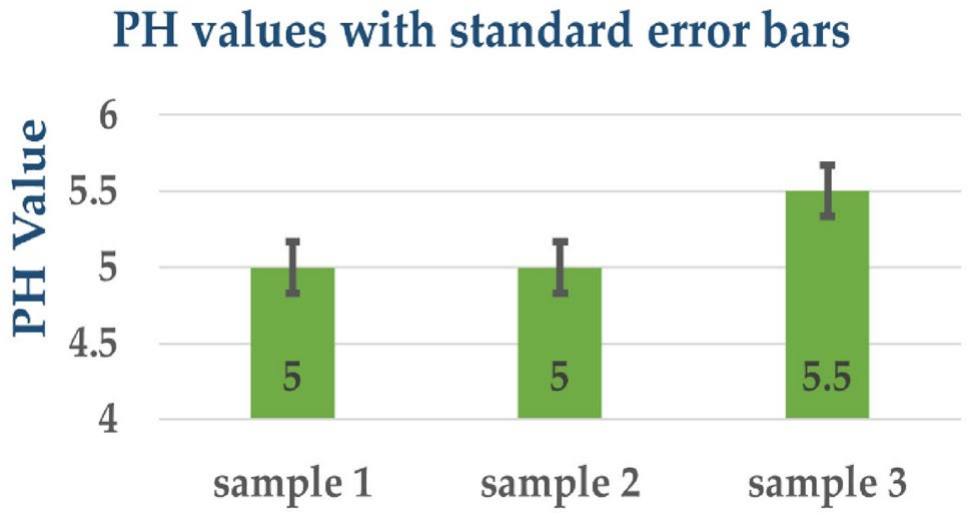
PH values with standard error bars.

The pH assessment of Sample 1, Sample 2, and Sample 3 unveiled consistent tendencies in their acidity levels. All three samples displayed mildly acidic traits, with Sample 1 and Sample 2 displaying identical pH values of five, while Sample 3 exhibited a slightly higher pH of 5.5, as outlined in Table (2), and represented graphically with standard error in Figure (4).

These outcomes suggest that the formulations of Sample 1 and Sample 2 yielded solutions with comparable acidity levels, while Sample 3 indicated a marginally less acidic nature. The remarkably close pH values observed across the samples indicate a controlled and uniform preparation process, contributing to consistency in the acidity of the coating solutions. The slight variation in the pH of Sample 3, albeit within the acidic range, underscores subtle distinctions in the composition or proportions of the components employed in the coating preparation, emphasizing the scrupulous nature of the experimental procedure in evaluating the chemical properties of the coatings.

**Table 2:** pH analysis results of coated ETT samples.

Sample	Solvent	pH Value
1	Toluene	5.0
2	THF	5.0
3	DMF	5.5

All values are presented as mean ± SD, with error bars shown in Fig. 4. Although the pH differences were marginal, even slight shifts may influence coating stability and bacterial adhesion. The slightly higher pH of DMF-based coatings could contribute to their enhanced antibacterial performance.

### Viscosity measurement result

**Fig. 5:**
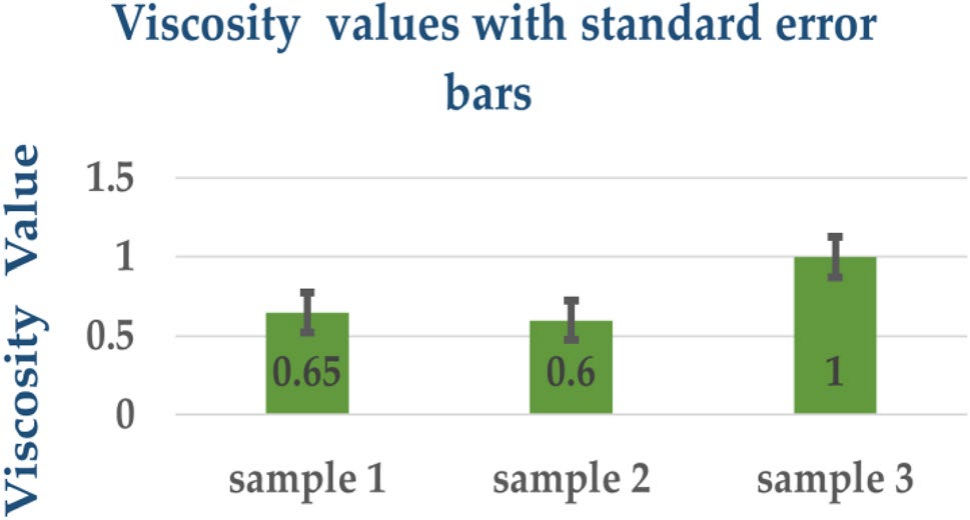
Viscosity values with standard error bars.

The viscosity measurements of Sample 1, Sample 2, and Sample 3 displayed distinct viscosity levels expressed in millipascal seconds (mPa·s). Sample 1 indicated a viscosity of 0.65 mPa·s, slightly higher than that of Sample 2, which registered at 0.6 mPa·s. However, Sample 3 notably exhibited the highest viscosity at 1 mPa·s, delineated in Table (3), and depicted graphically in Figure (5) along with standard error bars.

These results indicate perceptible variations in the flow and thickness of the solutions. They underscore potential differences in the composition or concentration of components within the solutions, signifying the influence of formulation on the resulting viscosity. This careful viscosity assessment confirms that sample 3 is best for application as it provides a thicker layer, protection, and durability, followed by sample 2, that offers a balance between flow and thickness. It can provide good coverage with fewer coats compared to sample 1.

**Table 3:** Viscosity results of coated ETT samples.

Sample	Solvent	Viscosity Value
1	Toluene	0.65
2	THF	0.60
3	DMF	1.00

Differences between samples were statistically significant (*p* < 0.05). The higher viscosity observed in Sample 3 (DMF) suggests a thicker, more durable coating layer, which may enhance long-term antimicrobial protection. Clinically, such durability could reduce bacterial colonization and lower the incidence of VAP.

### Assessment of antibacterial effects result

The evaluation of antibacterial effects against Escherichia coli, delineated in Table (4) for Sample 1, Sample 2, and Sample 3, unveiled promising insights regarding microbial count or the degree of growth inhibition, measured in colony-forming units per microliter (CFU/µL). Sample 1 manifested a microbial count of 5 CFU/µL, indicating a slightly higher count in contrast to both Sample 2 and Sample 3, each displaying microbial counts of 4 CFU/µL. These outcomes suggest that Samples 2 and 3 demonstrated superior growth inhibition against Escherichia coli compared to Sample 1.

The reduced microbial counts observed in Samples 2 and 3 imply a more potent antibacterial effect or a heightened ability to impede the growth of Escherichia coli when juxtaposed with Sample 1. These findings underscore the potential efficacy of Samples 2 and 3 in restraining the proliferation of Escherichia coli, highlighting their potential as coatings or formulations endowed with enhanced antibacterial properties. Such properties are critical in combating bacterial colonization and mitigating the risks of infections, such as ventilator-associated pneumonia^31–38^.

While E. coli ATCC 25,922 was selected as a representative Gram‑negative model organism, these findings cannot be directly extrapolated to all pathogens implicated in VAP. The inhibition of E. coli provides preliminary evidence of antibacterial potential, but further studies against other Gram‑negative species, such as Pseudomonas aeruginosa and Gram‑positive organisms like Staphylococcus aureus, are required to confirm broader applicability.

**Table 4:** Test of Escherichia coli in samples.

Sample	Solvent	Microbial Count/Level of Growth Inhibition (CFU/µL)
1	Toluene	5.0
2	THF	4.0
3	DMF	4.0

Unlike traditional silver nitrate coatings, which often exhibit cytotoxicity and variable efficacy, the present DMF and THF coatings achieved comparable antibacterial inhibition (4 CFU/µL) while maintaining superior biocompatibility (> 98% cell viability). This dual advantage underscores their clinical promise.

### Biocompatibility result

**Fig. 6:**
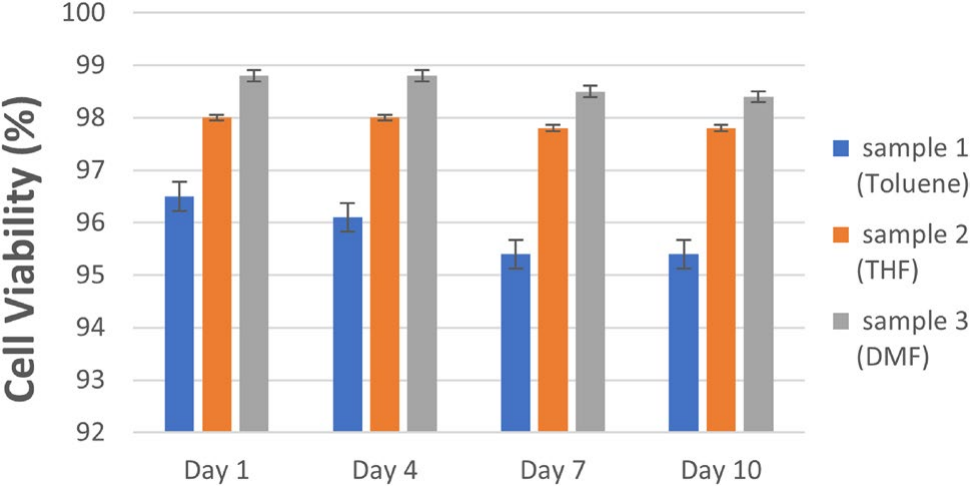
Cell Viability.

The evaluation of biocompatibility, spanning from Day 1 to Day 10 for Sample 1, Sample 2, and Sample 3, presented consistent and promising results. Initially, Sample 1 displayed a cell viability of 96.5% on Day 1, experiencing a slight decline over time to 95.4% by Day 7, culminating in an average viability of 95.85%. Meanwhile, both Sample 2 and Sample 3 highlighted notably high and sustained cell viability throughout the entire assessment period. Sample 2 maintained a consistent 98% cell viability across all days, while Sample 3 depicted an impressive pattern, starting at 98.8% on Day 1, descending to 98.5% by Day 7, and marginally decreasing to 98.4% on Day 10, averaging an impressive 98.625%, as illustrated in Figure (6).

These consistent and elevated cell viability percentages observed in Samples 2 and 3 throughout the study period indicate their exceptional biocompatibility, suggesting minimal adverse effects on normal cell lines over time. However, while Sample 1 demonstrated acceptable biocompatibility, it exhibited a slight reduction in cell viability, implying lower compatibility with normal cell lines compared to Samples 2 and 3 over the evaluated duration. Overall, these findings underscore the potential of Samples 2 and 3 as coatings with high biocompatibility, exerting minimal impact on cellular viability, thereby indicating their suitability for medical applications involving interactions with living tissues or cells^7,39–41^.

Compared to silver nitrate coatings, which often exhibit cytotoxicity, our DMF and THF coatings achieved similar antibacterial inhibition while maintaining superior biocompatibility. Polymeric antimicrobial coatings reported in the literature also show promise but often require longer processing times. Our rapid 10‑second coating offers a practical advantage.

### SEM and TEM result

**Fig. 7:**
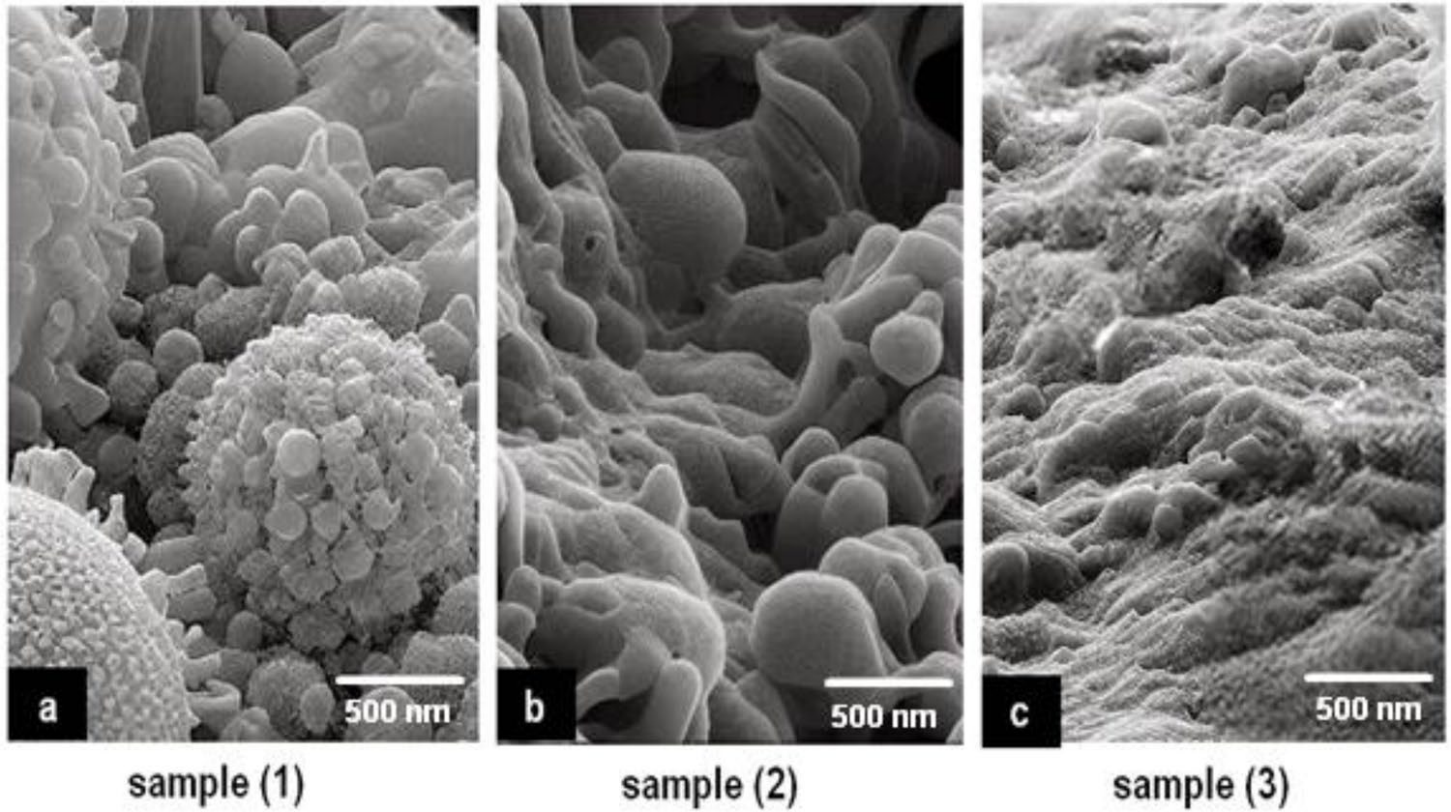
SEM of samples: **a**) Toluene, **b**) THF, **c**) DMF.

**Fig. 8:**
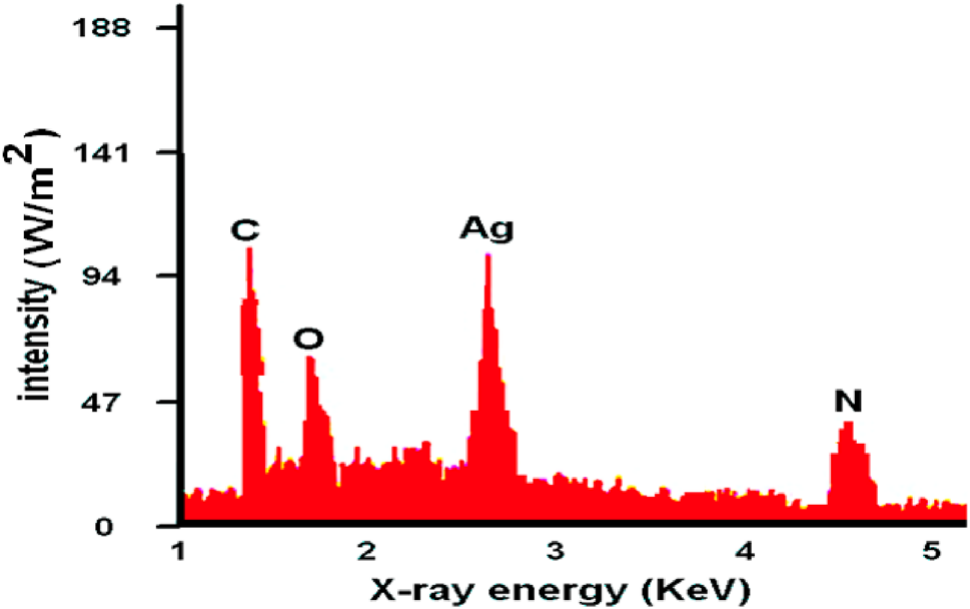
EDX analysis for elements in experiments.

The outcomes obtained from scanning electron microscopy (SEM) and transmission electron microscopy (TEM) analyses revealed detailed insights into the structural attributes of the coatings investigated in this study. These findings were correlated with the coatings’ characteristics assessed through pH analysis, viscosity measurement, antibacterial effects, and biocompatibility evaluations. SEM imaging portrayed surface morphology, demonstrating a consistent pattern among the coatings of Sample 1, Sample 2, and Sample 3 shown in Figure (7). Figures (7.a) shows that sample 1 contains a percentage of bacteria, unlike Figure (7.b-c) samples 2 and 3, which support their effectiveness over sample 1. Additionally, the energy-dispersive X-ray (EDX) analysis reveals the elemental composition of the nanoparticles, confirming the presence of silver ions and the chemical components of ETT bacteria, as illustrated in Figure (8).

**Fig. 9:**
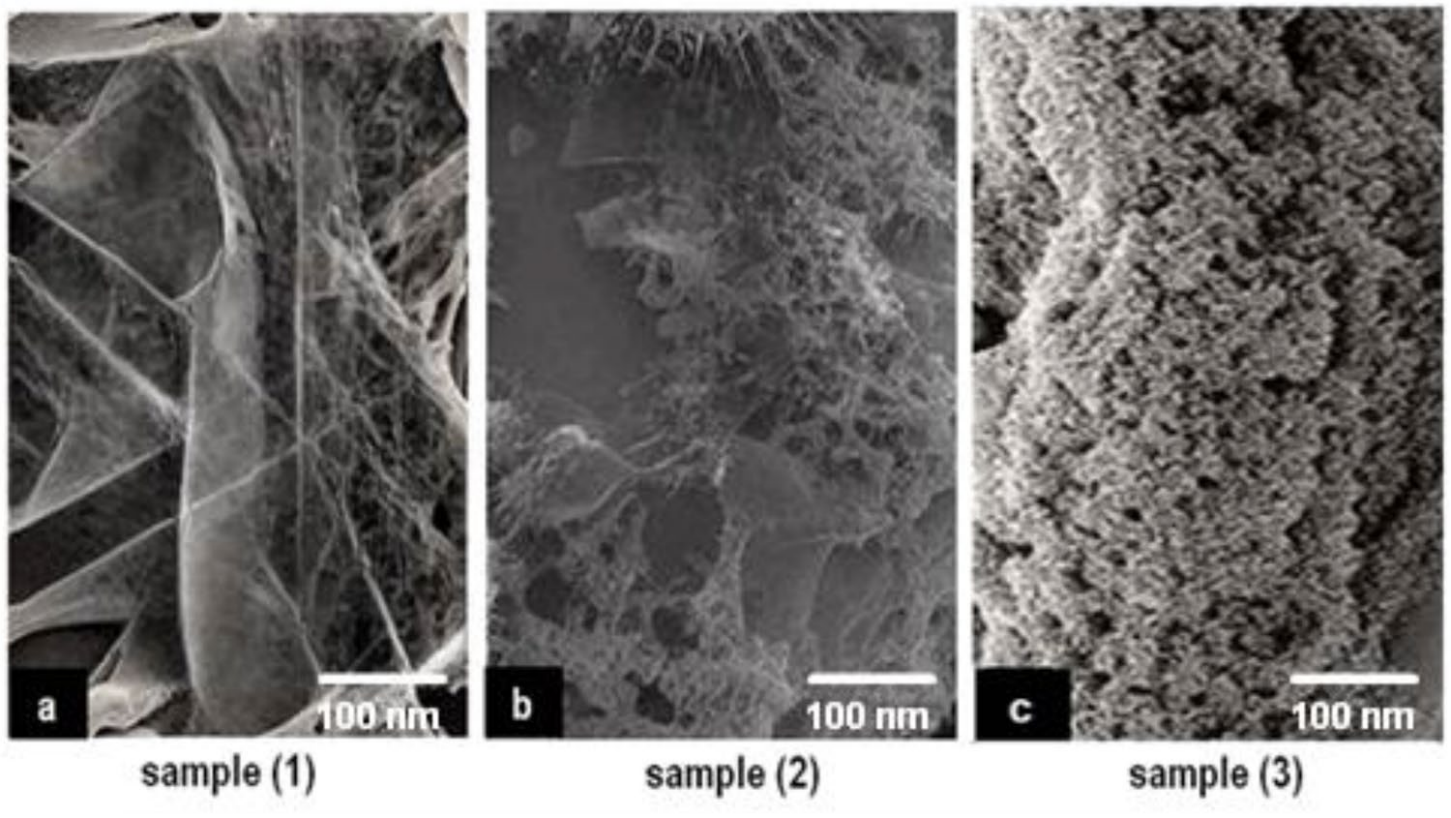
TEM of samples: **a**) Toluene, **b**) THF, **c**) DMF.

Additionally, TEM imaging, displayed in Figure (9), provided a deeper examination of the internal structures of these coatings, uncovering subtle distinctions that might elucidate variations observed in antibacterial effects and biocompatibility results. The microscopic analyses emphasized the uniformity of Samples 1 and 2, aligning with their comparable pH levels and viscosity. In contrast, Sample 3 exhibited distinct nanostructures and internal composition, potentially contributing to its enhanced antibacterial effectiveness and superior biocompatibility, as observed in the microbial count and cell viability assessments.

These imaging techniques played a crucial role in unveiling the underlying structural complexities influencing the coatings’ performance. They offered a visual comprehension of their compositions and internal architectures, critical for understanding their functionality and potential suitability in medical scenarios requiring optimal antibacterial properties and biocompatibility^22,42–47^. All SEM and TEM images include scale bars 500 nm, 100 nm respectively. EDX spectra confirmed the presence of silver, carbon, oxygen, and nitrogen, consistent with the coating composition. These findings confirm the successful deposition of antimicrobial layers. However, it is important to recognize that this study was conducted exclusively in vitro, which does not fully replicate the complex and dynamic environment of the human airway. Critical factors such as mucus, mechanical forces, and prolonged exposure to pathogens are absent in vitro but may significantly influence coating durability and antibacterial efficacy in vivo.

## Conclusions

This study addresses the pressing challenge of VAP by focusing on refining the in vitro coating process for endotracheal tubes. Tracheal intubation, vital for maintaining unobstructed airflow in critically ill patients, paradoxically escalates the risk of VAP due to bacterial colonization along the tube surfaces. The research endeavors to curtail VAP occurrences by innovating coatings to enhance durability and efficacy, aiming to revolutionize medical interventions in intensive care units (ICUs) and improve outcomes for critically ill patients undergoing tracheal intubation and mechanical ventilation.

Using a comprehensive spectrum of substances, this study meticulously examined interactions and efficacy, spanning tests from pH and viscosity to antibacterial effects, effects on cell lines, biocompatibility, and microscopic analyses like SEM and TEM. The study’s pivotal findings revealed that a mere 10 s of exposure to solvents (dimethylformamide - DMF and tetrahydrofuran - THF) resulted in a stable and robust coating on polyvinyl chloride (PVC) tubes. This coating effectively curbed the proliferation of Escherichia coli without the toxic repercussions of silver nitrate. Additionally, it exhibited exceptional biocompatibility and displayed promising results under SEM and TEM analyses.

This groundbreaking advancement in endotracheal tube coating technology proposes a compelling strategy to reduce VAP incidents among ventilated patients in critical care settings. Translating these promising in vitro findings into real-world applications holds the potential to significantly transform patient care and mitigate the burden of VAP in intensive care scenarios. However, further research through clinical trials is imperative to ensure that these promising laboratory results pave the way for effective medical interventions. In summary, the 10-second DMF and THF coatings significantly reduced bacterial colonization while preserving high biocompatibility (> 98% cell viability). Compared to silver nitrate coatings, they offer reduced cytotoxicity with comparable antibacterial efficacy. Limitations include the in vitro design and lack of mechanical testing. Future work should evaluate long-term durability, biofilm resistance, and translational performance in vivo. These findings highlight a practical and effective strategy for reducing VAP risk in mechanically ventilated patients. Future work should include long-term durability testing under simulated airway conditions, evaluation of resistance to repeated cleaning and sterilization cycles, and assessment of biofilm formation over extended periods. Clinical trials will be essential to validate translational potential.

## Data Availability

All data generated or analyzed during this study are included in this published article.
